# Canonical and Noncanonical Sites Determine NPT2A Binding Selectivity to NHERF1 PDZ1

**DOI:** 10.1371/journal.pone.0129554

**Published:** 2015-06-12

**Authors:** Tatyana Mamonova, Qiangmin Zhang, Jahan Ali Khajeh, Zimei Bu, Alessandro Bisello, Peter A. Friedman

**Affiliations:** 1 Department of Pharmacology & Chemical Biology, University of Pittsburgh School of Medicine, Pittsburgh, Pennsylvania, United States of America; 2 Department of Chemistry and Biochemistry, City College of New York, Chemistry and Biochemistry PhD program, CUNY, New York, New York, United States of America; 3 Department of Chemistry, City College of New York, New York, New York, United States of America; 4 Department of Structural Biology, University of Pittsburgh School of Medicine, Pittsburgh, Pennsylvania, United States of America; National Institutes of Health, UNITED STATES

## Abstract

Na^+^/H^+^ Exchanger Regulatory Factor-1 (NHERF1) is a scaffolding protein containing 2 PDZ domains that coordinates the assembly and trafficking of transmembrane receptors and ion channels. Most target proteins harboring a C-terminus recognition motif bind more-or-less equivalently to the either PDZ domain, which contain identical core-binding motifs. However some substrates such as the type II sodium-dependent phosphate co-transporter (NPT2A), uniquely bind only one PDZ domain. We sought to define the structural determinants responsible for the specificity of interaction between NHERF1 PDZ domains and NPT2A. By performing all-atom/explicit-solvent molecular dynamics (MD) simulations in combination with biological mutagenesis, fluorescent polarization (FP) binding assays, and isothermal titration calorimetry (ITC), we found that in addition to canonical interactions of residues at 0 and -2 positions, Arg at the -1 position of NPT2A plays a critical role in association with Glu43 and His27 of PDZ1 that are absent in PDZ2. Experimentally introduced mutation in PDZ1 (Glu43Asp and His27Asn) decreased binding to NPT2A. Conversely, introduction of Asp183Glu and Asn167His mutations in PDZ2 promoted the formation of favorable interactions yielding micromolar *K*_D_s. The results describe novel determinants within both the PDZ domain and outside the canonical PDZ-recognition motif that are responsible for discrimination of NPT2A between two PDZ domains. The results challenge general paradigms for PDZ recognition and suggest new targets for drug development.

## Introduction

**Fig 1:**
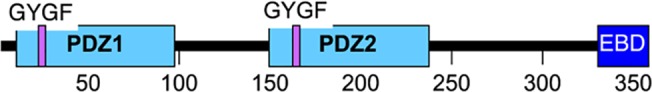
Schematic representation of NHERF1. NHERF1 possesses two tandem PDZ domains and a carboxy-terminal ezrin binding domain (EBD).

Na^+^/H^+^ Exchanger Regulatory Factor-1 (NHERF1), also known as the 50-kDa ezrin-binding protein EBP50, is a multi-domain scaffolding protein that coordinates the assembly and trafficking of transmembrane receptors and ion channels [1–3]. NHERF1 possesses two tandem PDZ (PSD-95/*Drosophila* disk large/ZO-1) domains of ~90 amino acids and an ezrin-binding domain (EBD), through which it binds the actin cytoskeleton (Fig 1). PDZ domains of NHERF1 recognize the X-S/T-X-Φ_COO_^-^ sequence of target partners (class I PDZ-binding motifs), where X is promiscuous and Φ is a hydrophobic residue. By convention, ligand residues are numbered backwards from zero at the carboxy terminus [4–8]. NHERF1 binds an extensive set of proteins including the parathyroid hormone receptor (PTHR), the β_2_-adrenergic receptor (β_2_-AR), the cystic fibrosis transmembrane regulator (CFTR), the P2Y1 receptor, and the thromboxane A_2_ receptor, among others, that harbor a PDZ ligand. These and most target substrates bind to PDZ1 or PDZ2 with more-or-less comparable affinity [9,10]. PDZ1 and PDZ2 of NHERF1 possess identical (GYGF) core-binding motifs [4,10–12] (Fig 1). Primary, or canonical, interactions occur through the GYGF core-binding motif of NHERF1 PDZ domains and the carboxy-terminal hydrophobic residue at ligand position 0. Another canonical interaction occurs between Ser/Thr at ligand position -2 and the structurally conserved His72 (PDZ1) or His212 (PDZ2) [4,10,13]. Despite the sequence and structural similarity of PDZ1 and PDZ2 a subset of ligands uniquely binds only PDZ1 or PDZ2. The type II sodium-dependent phosphate co-transporter (NPT2A, *SLC34A1*), for instance, binds only PDZ1 [10,14–16]. Hence, structure- and sequence-based algorithms [6,10,17–21] that have been advanced to predict PDZ-binding specificity are insufficient to explain instances of unique binding to PDZ1 or PDZ2. Recent observations suggest that extended sequences beyond the canonical PDZ domain fold, as well as outside the short carboxy-terminal motifs of target ligands, can partially address these shortcomings [4,10–13,22,23]. Clearly, additional structural determinants and interactions distant from the core-binding motif differentiate sequence recognition of the two PDZ domains of NHERF1. The goal of the present study was to determine and characterize the binding factors that confer specificity of NPT2A to PDZ1. We applied a two-pronged approach of molecular dynamics simulation and experimental measurements.

The three-dimensional structures of ligand-bound isolated PDZ domains provide the key atomic details about the binding interface and insights into the mechanism of complex formation. Few X-ray and NMR structures of the NHERF1 PDZ domains with peptides mimicking the PDZ-binding motifs of target ligands are available [4,10–13,22]. Most investigations address the interaction between the core residues forming the PDZ domain binding site with the four or five terminal amino acids of the ligand PDZ-recognition motif [11,13]. However, specificity of the interaction can be modulated by extended and remote binding determinants of the PDZ domains with carboxy-terminal ligand residues [10,19,22,23]. Here we sought to determine noncanonical elements outside the core-binding motif and beyond the formal carboxy-terminal PDZ ligand that are responsible for the specificity of interaction between PDZ1 domain of NHERF1 and NPT2A. By performing extensive all-atom molecular dynamics (MD) simulations of the 22-residue carboxy-terminal tail of NPT2A and NHERF1 PDZ domains we identified the specific determinants of PDZ1-NPT2A interactions. Based on these findings, we then introduced in PDZ2 the residues discovered in PDZ1 predicted to be required for NPT2A binding. These mutations now conferred NPT2A binding on PDZ2. The combined approach of computational modeling and experimental testing allowed prediction of the structural determinants and unique interactions underlying the PDZ1-NPT2A complex formation.

## Results

### Structure and dynamics of the PDZ1-NPT2A complex

**Fig 2:**
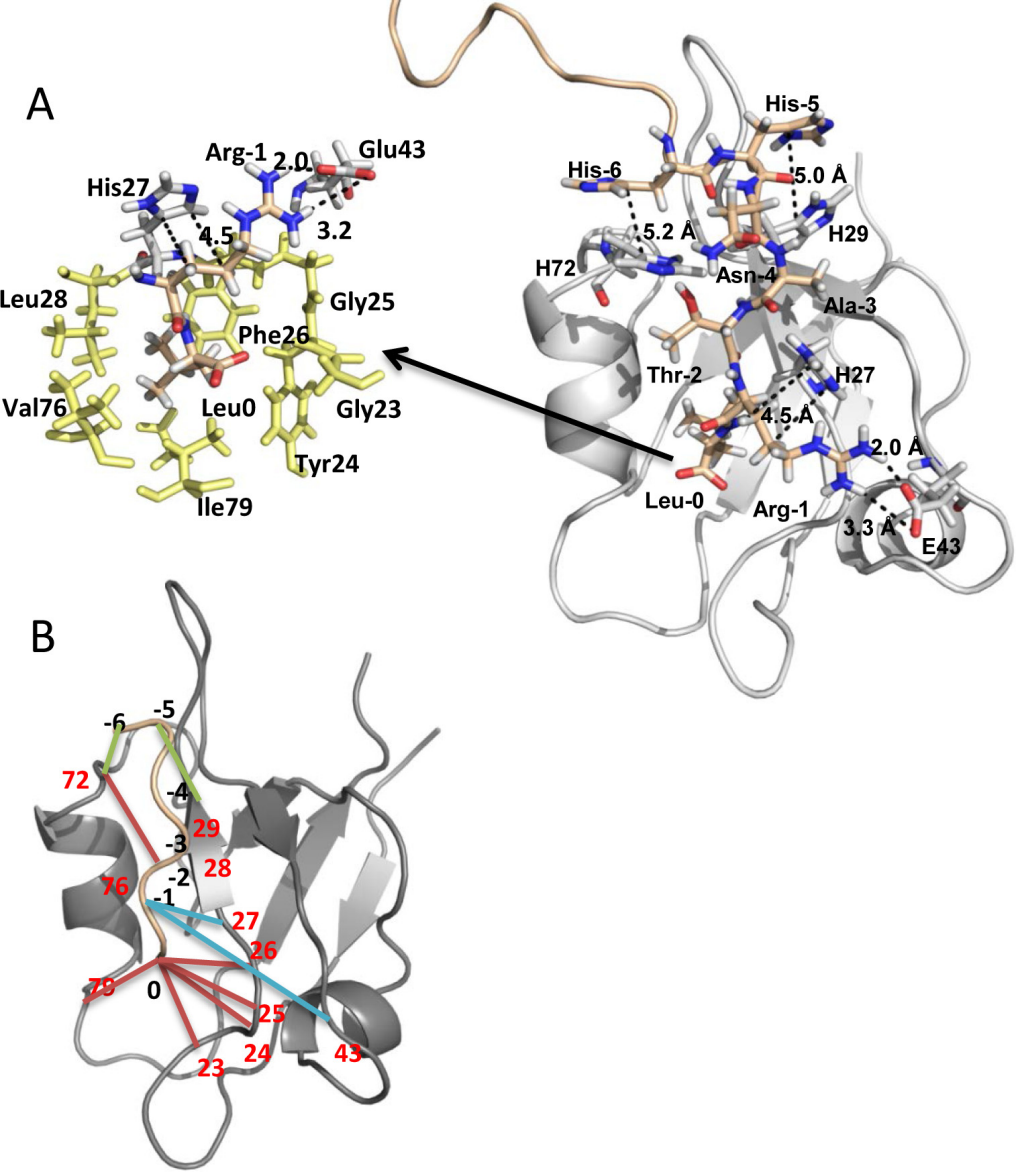
Model structure of PDZ1 in complex with the NPT2A peptide. (**A**) PDZ1 and NPT2a are shown in grey and wheat, respectively. The NPT2A peptide is shown in stick representation and numbered from 0 to -6. Residues forming the canonical hydrophobic pocket of PDZ1 are shown in yellow (stick representation). A salt bridge between the carboxylate group of Glu43 and the guanidino group of Arg^-1^ of NPT2A, carbon-carbon interactions between His27 and Arg^-1^ as well as hydrophobic interactions between His29 and His^-5^, and His72 and His^-6^ are shown as black dotted lines. Hydrogen atoms are white, oxygens red, and nitrogens blue. (**B**) Positions pairs that predict NPT2A selectivity for PDZ1 are illustrated. The orange lines indicate the pairs involved in canonical interactions. The blue and green lines indicate the pairs involved in noncanonical interactions. Residue positions in the PDZ1 domain and NPT2A are highlighted in red and black, respectively.

The core-binding residues of PDZ1 that recognize the limited carboxy-terminal motif-NATRL of NPT2A were suggested by our prior molecular dynamics simulation study [24] (S1 Fig). To elucidate the features responsible for PDZ1 binding specificity of NPT2A and characterize the structural determinants of the NPT2A recognition we performed the extensive MD simulations of PDZ1 bound to the carboxy-terminal 22-residue peptide of NPT2A. A representative snapshot from the MD simulation is shown in Fig 2A and referred to as the wild-type (WT) PDZ1-NPT2A complex. To monitor the stability of the system during MD simulations, we first performed an equilibration run that analyzed the root mean square deviation (RMSD) of Cα atoms of PDZ1 domain (residues 13–91) relative to the starting structure. The average RMSD value along the equilibration phase was 1.1 ± 0.1Å. The RMSD remains stable between 1.1 and 1.4 ± 0.1Å over the production MD simulation.

The binding of the NPT2a ligand does not cause conformational changes of the binding pocket of PDZ1. The average RMSD value of the Cα atoms of residues formed a binding pocket (Gly23, Tyr24, Gly25, Phe26 (GYGF loop), Leu28, Val76 and Ile79) was 0.47Å and 0.55Å throughout the equilibration phase and production simulation, respectively.

We also calculated the RMSDs of the backbone atoms for the ligand residues located in the binding pocket (position 0 to -4) relative to their starting position. Small fluctuations in the range of 0.6–1.4Å during the first 25 ns of the equilibrium MD simulation were observed. The five carboxy-terminal residues of the NPT2A peptide then reach a stable conformation with an average RMSD value of the backbone atoms of 0.9 ± 0.1Å over the entire MD trajectory. The absence of backbone conformational changes for the core of PDZ1, as well as for the carboxy-terminal motif of the bound peptide during equilibration and production simulations is evident from the low RMSD values and indicates that the resulting complex is stable and remains close to the initial structure.

The local mobility of each protein residue obtained from the RMSF calculation of the Cα atoms with respect to the starting structure throughout the trajectory is illustrated in S2 Fig. The result suggests that the structure of PDZ1 rather rigid. RMSF values increase up to 3 and 5Å for the N- and C-terminal regions. High RMSF values are displayed by turns and loops. Flexibility of residues from the carboxylate-binding loop and β2 sheet (residues 23–29) creates a favorable binding pocket to accept the carboxy-terminal Leu of NPT2A. The β2-β3 loop (residues 31–35) is flexible and therefore may accommodate bulky amino acid residues after ligand position -4. With the exception of the β2-β3 loop (residues 31–35) and carboxylate-binding loop (residues 19–23), PDZ1 bound NPT2A displays rather low RMSF values (RMSF < 1Å). The comparative rigidity of PDZ1-bound peptide is corroborated by the analysis of canonical and specific interactions that are observed in the MD simulation.

According to the MD model, the hydrophobic side chain of Leu^0^ settles deep in a hydrophobic cavity formed by the side chains of Tyr24, Phe26, Leu28 of the β2 sheet, and Val76 and Ile79 of the α2-helix (Fig 2A and S1 Fig). The side chain conformations of these residues are in a favorable orientation to form both hydrophobic contacts as well as hydrogen bonds with Leu^0^ (Fig 2A and S3 Fig). Another conserved interaction is formed between the imidazole group of His72 at the top of the α2-helix and the OH group of Thr^-2^. The high probability of canonical interactions involved Leu^0^ and Thr^-2^ along the MD simulation (Fig 2B and S1 Table) is in strong agreement with the conserved contacts observed by X-ray and NMR and provides a structural basis for the recognition of the carboxy-terminal motif of target ligand [10,24]. Structural superposition of the average structure of the PDZ1 domain from the MD simulation and X-ray structure (PDB code: 1GQ4) is presented in S2 Fig. Both structures show similar backbone conformations with an RMSD value of 1.1Å (residues 13–91). Also, the side chain conformations of residues forming the binding groove (Phe26, Leu28, Ile79 and Val76) are very similar. The side chain of Tyr24 shows a small rotation toward the carboxy-group of Leu^0^ compared to the X-ray structure.

### Specific Binding determinants for the PDZ1–NPT2A complex

A major interest for us is specific binding determinants which may explain the selectivity of PDZ1 for NPT2A. These determinants directly contact target ligand and locate beyond the hydrophobic cavity of PDZ1 (Fig 2B). For instance, Glu43 from the first α-helix (αA) of PDZ1 is involved in the electrostatic interaction with Arg^-1^ of NPT2A (Fig 2A) [24]. The 100 ns MD simulations permit detailed evaluation of the formation and dynamics of this interaction. During the first 15 ns of the MD simulation we observed rotation of the side chain of Arg^-1^ toward the carboxylate group of Glu43. The carboxyl oxygen atoms (Oε^1^ and Oε^2^) of Glu43 were subsequently close to the guanidino group of Arg^-1^ and form electrostatic interactions. During the next 15 ns of the MD simulation, the distance between two charged groups stabilizes between 2Å and 3Å and remains stable along the rest of the simulation. Analysis of non-covalent interactions predicts formation of a bifurcated salt bridge between the carboxylate oxygens (Oε^1^ and Oε^2^) of Glu43 and the NHη^2^ group of Arg^-1^ during the course of the simulation (Fig 2A and S1 Table).

MD simulations performed here reveal a novel and specific role of His27 for the formation of an electrostatic interaction between Glu43 and Arg^-1^. Analysis of the orientation of His27 shows that the imidazole ring faces toward the side chain of Arg^-1^ (Fig 2A). The ring Cδ^2^ atom forms a hydrophobic interaction with the Cβ atom of Arg^-1^. The ring Cε^1^ atom is in hydrophobic contact with the Cγ atom of Arg^-1^ (Fig 2A and S1 Table). The distance between the C-C pairs stabilizes after approximately 20 ns of MD simulation and varies between 4–5Å along the balance of the MD simulation. Thus, His27 may provide local stability by facilitating salt bridge formation between the positively charge guanidino group of Arg^-1^ near the negatively charged carboxylate group of Glu43.

**Fig 3:**
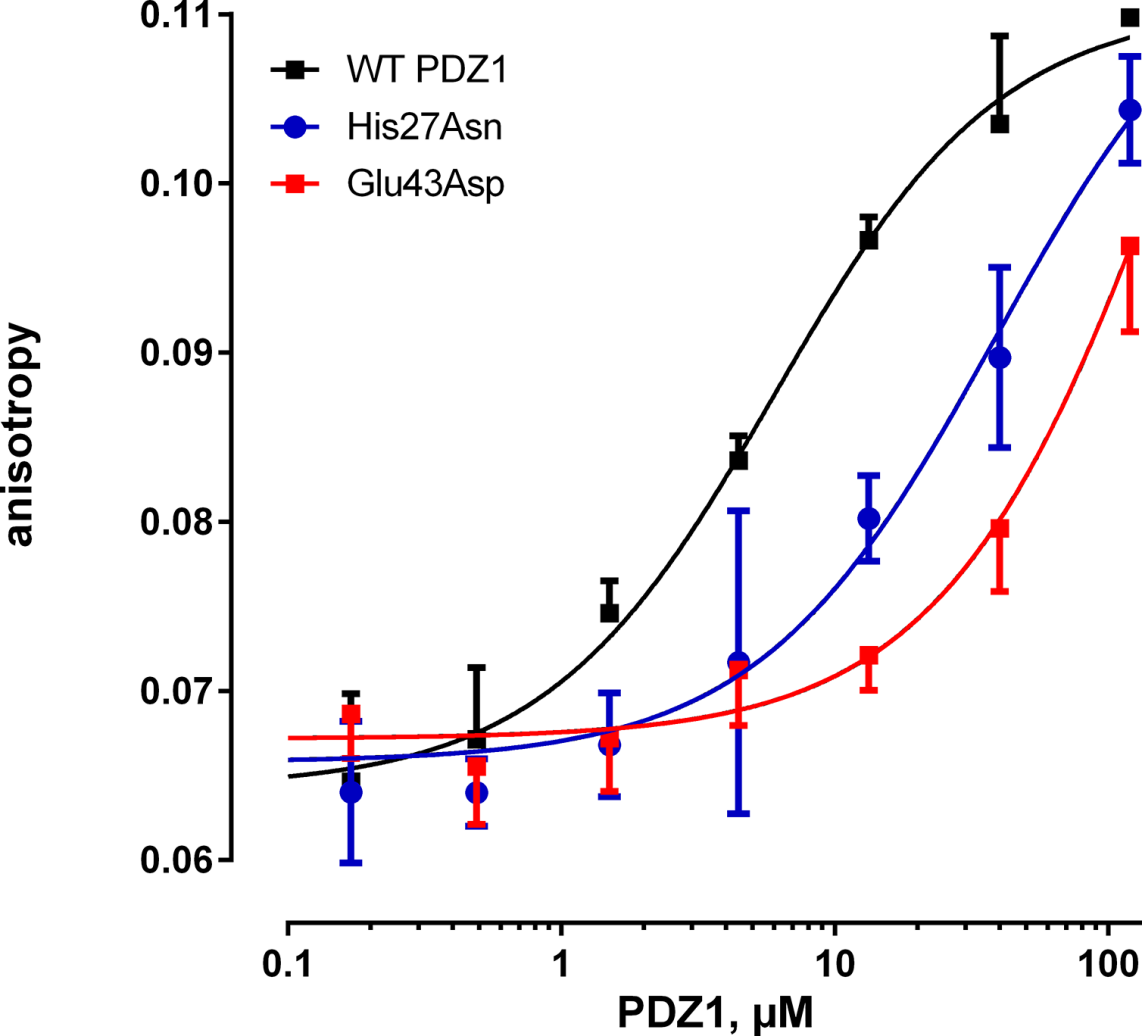
Fluorescent polarization binding studies of the modified PDZ1 domain. Representative fluorescence anisotropy binding curves for the labeled NPT2A peptide (1 μM) to WT PDZ1 (11–120), His27Asn (11–120), or Glu43Asp (11–120) PDZ1 mutants of NHERF1 are shown.

To validate the computational results that His27 and Glu43 of PDZ1 are essential for NPT2A binding, we generated recombinant PDZ1 with His27Asn and Glu43Asp mutations and measured their effect on NPT2A peptide binding by FP (Table 1) and ITC (Table 2). WT PDZ1 interacts with the NPT2A peptide with a *K*_D_ of 3.1–5.5 ± 0.6 μM, whereas both Glu43Asp and His27Asn mutations decrease the interaction with NPT2A (Fig 3, Table 1 and Table 2) confirming the predictions from the modeling. By applying the relation (Eq 3), we calculated the free energy (ΔG°) and evaluated the entropy (ΔS°) using Eq 4 (Table 2).

**Table 1:** Binding affinity of NHERF1 PDZ constructs and NPT2A measured by FP.

PDZ construct	*K*_D_/EC_50_, μM^a^
PDZ1 (1–140)	1.7 ± 0.2
PDZ1 (1–140) pH 5.5	16.1 ± 3.3
PDZ1 (1–140) pH 6.0	8.0 ± 1.8
PDZ1 (11–120)	5.5 ± 0.6
PDZ1 H27N (11–120)	N/D^a^
PDZ1 E43D (11–120)	N/D
PDZ2 (133–300)	N/D
PDZ2 D183E	20.0 ± 10
PDZ2 N167H/D183E	11.7 ± 2.0

^a^ N/D No detectable binding

**Table 2:** Binding affinity of NHERF1 PDZ1 constructs and NPT2A measured by ITC.

PDZ1 construct	*K*_a_, μM	ΔH^o^, kcal/mol	ΔS^o^,cal/mol/K	ΔG^o^,kcal/mol	N
PDZ1 (1–120)	3.1 ± 0.3	-8.2± 0.8	-2.1±0.2	-7.6±0.8	0.92
PDZ1 H27N (1–120)	5.3 ± 0.5	-7.5±0.7	-0.86±0.08	-7.2±0.7	0.61
PDZ1 E43D (1–120)	10.4 ± 0.8	-3.0±0.3	12.7±1.0	-6.8±0.7	0.60
PDZ1 H27N/E43D (1–120)	17.9 ± 0.9	-5.8±0.6	2.3±0.3	-6.5±0.6	0.60

Notably, His27Asn and Glu43Asp effectively convert these residues to their naturally occurring counterparts in PDZ2, where the positions occupied by His27 and Glu43 in PDZ1 are Asn167 and Asp183 in PDZ2. Thus, both mutations alter the sequence of PDZ1 to resemble that of PDZ2.

### Interaction between the double PDZ2 mutant and NPT2A

**Fig 4:**
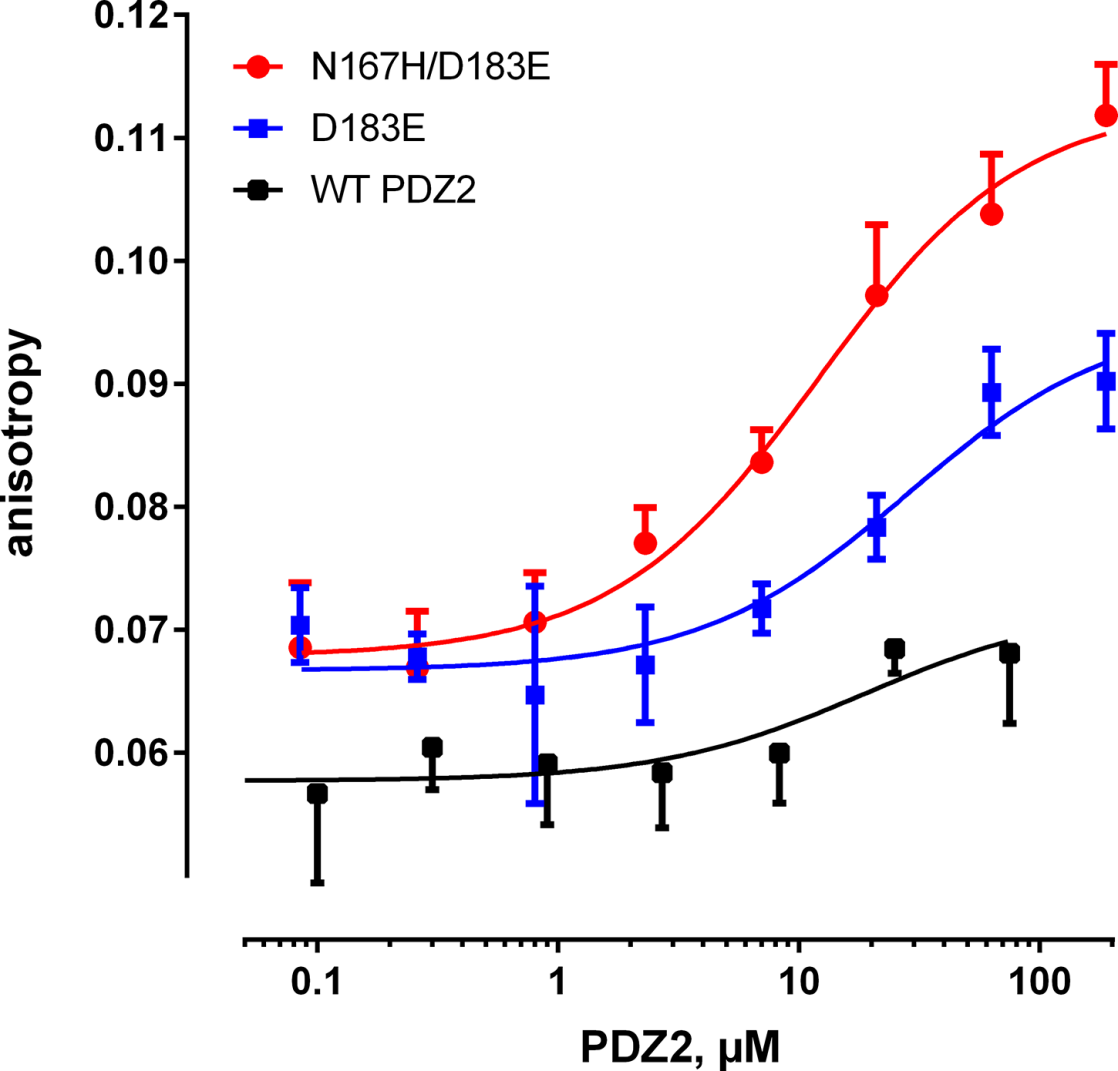
Fluorescent polarization binding studies of the modified PDZ2 domain. Representative fluorescence anisotropy binding curves for the labeled NPT2A peptide (1 μM) to WT PDZ2, Asp183Glu, or Asn167His/Asp183Glu PDZ2 mutants of NHERF1 are shown.

We reasoned that if the specificity of NPT2A binding to PDZ1 was attributable to the presence of His27 and Glu43 (Fig 2A), then mutating Asn167 to His (Asn167His) and Asp183 to Glu (Asp183Glu), that is, introducing in PDZ2 the key residues from PDZ1 (S3 and S4 Figs), should now permit NPT2A to bind PDZ2. To test this idea we generated recombinant Asp183Glu PDZ2 alone or in combination with Asn167His. The binding affinity of Asp183Glu and Asn167His/Asp183Glu to the NPT2A peptide was measured by FP. The Asp183Glu mutant interacts with the NPT2A peptide with micromolar affinity (Fig 4 and Table 1). The combined effect of the double mutation (Asn167His/Asp183Glu) may explain the further enhancement of the binding affinity for NPT2A (Fig 4 and Table 1).

To explore interactions between the double mutant and the NPT2A peptide, MD simulation was performed over 150 ns. MD simulation showed that the double PDZ2 mutant engages the NPT2A peptide at virtually identical binding site residues as in PDZ1 (S4 and S5 Figs). The peptide clearly maintains the same binding orientation in the double PDZ2 mutant as in WT PDZ1 (Fig 2A and S5 Fig). The NPT2A peptide now establishes interactions with the residues from the carboxylate binding loop (GYGF), α2-helix and β2 sheet of PDZ2, as well as forms a salt bridge with Asp183Glu (S2 and S3 Figs), functionally equivalent to the PDZ1-NPT2A complex (Fig 2A). We monitored the distance between the carboxylate group of Asp183Glu and the guanidino group of Arg^-1^ (S6A Fig) along the MD simulation. The distance is stable after approximately 30 ns of the MD simulation and reflects the formation of a salt bridge (S6A and S6B Fig). Our result also indicates that hydrophobic contacts between Asn167His and Arg^-1^ occur through the ring Cδ^2^ and Cε^1^ atoms of Asn167His and the Cβ and Cγ atoms of Arg^-1^, respectively (S6B Fig). After approximately 30 ns of MD simulation both distances stabilized between 4–5Å. The average values of 4.6 ± 0.6Å and 4.5 ± 0.5Å along the last 40 ns of MD simulation was calculated for the Cδ^2^-Cβ and Cε^1^-Cγ pairs, respectively.

The MD simulation was repeated with the shorter–NATRL sequence of the NPT2A peptide to verify the formation of a salt bridge between Asn183Glu and Arg^-1^ (see Supporting Information for details). The MD simulation results revealed that the–NATRL peptide binds the double PDZ2 mutant in an orientation and conformation similar to those identified for the NPT2A peptide (S5 Fig). As before, we observed a strong tendency for the formation of electrostatic interactions between the carboxylate group of Asp183Glu and the guanidino group of Arg^-1^ as well as hydrophobic contacts between the ring Cδ^2^ and Cε^1^ atoms of Asn167His and the Cβ and Cγ atoms of Arg^-1^, respectively. We also modeled the interaction between WT PDZ2, the single Asp183Glu PDZ2 mutant, and the–NATRL ligand. Notably, the limited–NATRL ligand was released from the PDZ2 binding pocket of both the WT PDZ2 and the single Asp183Glu PDZ2 mutant after approximately 30 ns of MD simulation.

### His-His interactions in PDZ1-NPT2A

Rotation of the His^-5^ side chain and formation of orientated stacking with His29 was observed during the first 40 ns of the MD simulation. Further calculations reveal that the imidazole ring of His^-5^ is close to the imidazole ring of His29 (Fig 2A) with an average ring centroid-centroid distance of 5.0 ± 0.3Å over the course of the MD simulation. We observed a stable parallel stacking arrangement of the imidazole ring of His^-5^ over the ring of His29 with the average angle between the normal vectors of two ring planes of 160 ± 20°. The MD simulations also showed that the side chain of His^-5^ could potentially attract the carboxylate group of Glu31, as well as the positively charged guanidino group of Lys32 from the β2-β3 loop. However, due to fluctuations of the carboxylate group of Glu31 as well as the guanidino group of Lys32, the bond length between the potential donor-acceptor pairs often exceeded 3.5Å.

Analysis of the MD simulation data predicts that the imidazole ring of His^-6^ rotates toward the imidazole ring of His72 during the first 15 ns of the simulation. The distance and angle between two rings then does not change conspicuously through the remainder of the simulation. An average ring centroid-centroid distance computed along the equilibrium MD simulation trajectory, yields an average value of 5.2 ± 0.3Å with an average angle between the normal vectors of two ring planes of 160 ± 10°. The favorable distance and angle between His^-6^ and His72 as well as His^-5^ and His29 strongly indicates a formation of stacking imidazole-imidazole interactions [24–26].

To determine the impact of the His residues at position -5 and -6 on the stability of the PDZ1-NPT2A complex, we computationally replaced these residues with Ala. MD simulations of the modified system (see Supporting Information for details) showed that substitution of Ala for His destabilizes the NPT2A peptide. The calculated RMSF of the Cα atoms per residue of the WT complex and for the modified system is presented in S7 Fig. We did not observe significant fluctuations over the MD simulation for WT PDZ1-NPT2A, whereas the Ala variants display higher RMSF values, especially for the loop regions. The major contribution to the higher RMSF comes from the loop between α2 and β5 (residues 61–78), partial β2 strand and β2-β3 loop (residues 27–35) as well as partial β3 and α1-helix (residues 40–47). Also, the Ala substitution imparts greater backbone flexibility, which increases the RMSF for the peptide residues at position -2, as well as upstream of position -8 (S7B Fig).

**Fig 5:**
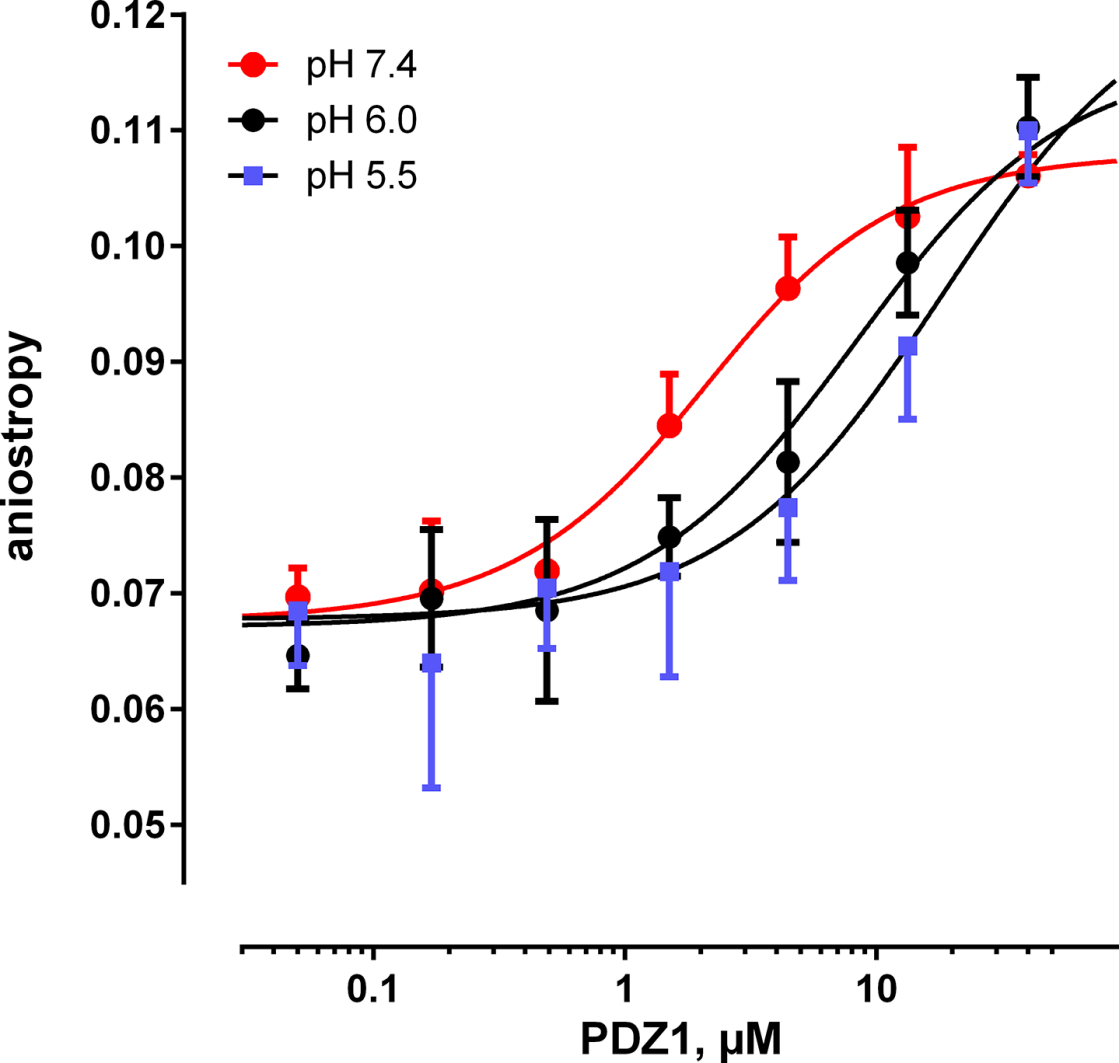
pH Dependence of PDZ1 binding to NPT2A. Representative fluorescence anisotropy binding curves for the labeled NPT2A peptide (0.5 μM) to WT PDZ1 (1–140) at pH 7.4; 6.0 and 5.5 are shown.

The p*K*_a_ values of His^-5^ and His^-6^ of NPT2A and His27, His29, and His72 of PDZ1 were estimated using PROPKA 3.1 [27] (S2 Table). We reasoned that if His-His interactions contribute to stabilizing the PDZ1-NPT2A interaction, then decreasing the pH should diminish the strength of these interactions. FP binding experiments performed at an acidic pH confirmed that the binding affinity between the PDZ1 domain and the NPT2A peptide is significantly reduced compared to pH 7.4 (Fig 5 and Table 1).

## Discussion

The two PDZ domains of NHERF1 share extensive similarity and identical GYGF core-binding motifs. Nonetheless, PDZ1 interacts with a larger and more diverse set of ligands compared to PDZ2 [9,10,28,29]. A far smaller subset of ligands exhibit preferential or unique binding to PDZ1 or PDZ2. The basis for this specificity is unknown and may hold considerable potential for understanding the basis of PDZ-ligand binding and targeting novel compounds to these sites. The primary goal of the present study was to identify the structural determinants that confer selective binding of NPT2A to PDZ1 of NHERF1.

MD simulations provide an atomic-level description of the principal interactions involved in assembling the PDZ1-NPT2A complex. The results predicted that a salt bridge between Glu43 and Arg^-1^ imparts a large stabilizing effect on PDZ1-NPT2A binding. The enthalpy (∆∆H^o^ = 5.2 kcal/mol) and entropy (∆∆S^o^ = 14.8 cal mol^-1^ K^-1^) changes for Glu43Asp PDZ1-NPT2A (Table 2) are consistent with this observation. The stability of the PDZ1-NPT2A complex decreases by ∆∆G^o^ = 0.8 kcal/mol (∆∆G^o^ = ∆G^o^_E43D_ - ∆G^o^_WT_) if this salt bridge is disrupted.

The presence of His27 was also projected to be required for stabilizing the PDZ1-NPT2A complex. The difference in ∆∆G^o^ (∆∆G^o^ = ∆G^o^_H27N_ - ∆G^o^_WT_) caused by mutation of His to Asn is estimated to be about 0.4 kcal/mol (Table 2). ∆∆H^o^ = 0.7 kcal/mol (∆∆H^o^ = ∆H^o^_H27N_ - ∆H^o^_WT_) and ∆∆S^o^ = 1.3 cal mol^-1^ K^-1^ (∆∆S^o^ = ∆S^o^_H27N_ - ∆S^o^_WT_) are consistent with our prediction that hydrophobic contacts formed between the ring C-atoms of His27 and Cβ and Cγ atoms of Arg^-1^ restrict the dynamic behavior of the side chain of Arg^-1^ and orientate the positively charged guanidine group of Arg^-1^ near the negatively charged side chain of Glu43. The formation of the His27Asn/Glu43Asp PDZ1-NPT2A complex is enthalpically (the dominant contribution of hydrogen bonds) and entropically (loss of hydrophobic contacts) unfavorable. The difference in the free energy of binding (∆∆G^o^) upon replacement of His27 by Asn and Glu43 by Asp is 1.1 kcal/mol. FP experiments performed for H27N and E43D PDZ1 mutants confirmed that the presence of both residues is essential for NPT2A binding (Fig 4). Together, our results point to a critical role of Glu43 and His27 for PDZ1-NPT2A binding. We propose that these residues uniquely stabilize the binding of NPT2A and define the specificity of the PDZ1 domain.

PDZ2 harbors Asn167 and Asp183 at the positions corresponding to His27 and Glu43 in PDZ1. MD simulations of PDZ2 with the bound–NATRL peptide predict that the side chain of Asp183 is too short to form an electrostatic interaction with Arg^-1^. The analysis of non-covalent interactions showed that Asn167 does not interact with the–NATRL peptide. The side chain of Asn167 is polar and preferentially surrounded with water molecules rather than establishing interactions with the ligand. Thus, the MD simulation does not predict an impact of the side chains of Asn167 and Asp183 on the PDZ1-NATRL binding, whereas the side chains of His27 and Glu43 establish stable interactions with the ligand. The computational predictions agree well with the binding experiments, showing only a very weak interaction between PDZ2 and the NPT2A peptide (Fig 4), whereas PDZ1 binds NPT2A with a *K*_D_ of 5.5 μM [22] (Fig 3 and Table 1). We reasoned that if the limited binding of NPT2A to PDZ2 stems from these structural considerations, then the conservative replacement of Asp183 with Glu, and Asn167 with His should then impart NPT2A binding to PDZ2. Our modelling analysis established that the double PDZ2 mutant (Asn167His/Asp183Glu) interacts with NPT2A in a structurally similar manner to the naturally occurring PDZ1-NPT2A complex (S3 and S4 Figs). The longer side chain of Asp183Glu forms an electrostatic interaction with the side chain of Arg^-1^ (S5 Fig) compared to the shorter side chain of Asp183. The imidazole ring C-atoms of Asn167His form multiple hydrophobic contacts with the Cβ and Cγ atoms of Arg^-1^, similar to those found in the PDZ1-NPT2A complex. These predictions were borne out by the biochemical experiments, where the single (Asp183Glu) and double mutations (Asn167His/Asp183Glu) in recombinant PDZ2 stabilized the NPT2A peptide in the binding site with EC_50_s of 20.0 and 11.7 μM, respectively. The notable difference in the binding affinity between the double mutant and WT PDZ2 strengthens the conclusion that His27 and Glu43 are essential for NPT2A binding. Furthermore, these two residues differentiate the binding properties of NHERF1 PDZ domains for the NPT2A ligand and explain the observed binding specificity of PDZ1 for NPT2A.

The computational and experimental results allowed us predict the role of His-His interactions in the formation of the PDZ1-NPT2A complex. Based on our working model we theorized that His^-5^ and His^-6^ form hydrophobic interactions with His29 and His72 of PDZ1, respectively, and, therefore, may be necessary to stabilize the PDZ1-NPT2A complex. The His^-5^His^-6^ -alanine substitution indicates that alanine residues do not interact with NPT2A and destabilize the peptide in the binding site and beyond compared to the WT system. The *K*_D_ values measured by FP at acidic pH 5.5 and pH 6.0 suggest that interactions between the protonated histidine pairs are unfavorable compared to physiological pH. At pH 5.5, the population of the protonated form of His^-5^ and His^-6^ (p*K*_a_ of 6.11 and 5.83, respectively) of NPT2A is high. Under these conditions we assume that His^-5^ tends to be far from His29 (p*K*_a_ of 5.98) due to the electrostatic repulsion of their positive charges. We further speculate that when the side chain of His^-6^ is protonated, the fraction of the protonated His72 is very small (p*K*_a_ of 4.90). The side chain of His72 is involved in the canonical interaction with the ligand residue at position -2 (Thr in the case of NPT2A) [4,24] and is unlikely to be protonated under these conditions. If the interaction between PDZ1 and NPT2a occurs near the apical membrane, where the pH is 6.3–6.9 [30], then the probability is high that His27 (p*K*_a_ of 6.49) may be protonated. Overall, the measured binding affinities at pH 5.5, 6.0, and 7.4 suggest decreased binding affinity between PDZ1 and the NPT2A peptide under acidic conditions. Thus, PDZ1 may explore the ionization behavior of the histidine residues at different pHs (6.0 ≤ pH ≤ 7.4) as a proton sensor to initiate association or dissociation of target ligands in the cell environment, where the cytoplasmic pH is 7.4–7.5 and the endosomal pH is 6.3 or less [31,32].

Notably, these two His residues are a unique feature of the NPT2A carboxy-terminal motif that is not found in other NHERF1 target ligands. In this respect the PDZ1-NPT2A complex differs significantly from other PDZ-ligand binary complexes, and the His residues may play a critical role in NHERF1-NPT2A recognition. We note that for the double PDZ2 mutant (Asn167His/Asp183Glu) bound NPT2A stacking between His-His pairs was not observed. We conjecture that natural mutation of Gly28 and Thr71 in PDZ1 to Ser168 and Gln211 in PDZ2 may screen His-His interactions. Future experimental work will be necessary to elucidate the role of His^-5^ and His^-6^ on PDZ1-NPT2A binding.

In summary, we applied a combined approach involving MD simulation with site-specific mutagenesis of recombinant proteins and biochemical measurements to identify structural determinants that define binding specificity of PDZ1 to NPT2A. The MD simulations and experimental results reveal that Glu43 and His27 control the interaction between PDZ1 with NPT2A. To verify that the presence of these features is critical for the NPT2A recognition we experimentally introduced single (Asp183Glu) and double mutations (Asn167His/Asp183Glu) that conferred binding of the NPT2A peptide to PDZ2 with micromolar affinity. Our study demonstrates that the PDZ1-NPT2A binding is pH dependent and may be regulated by His-His interactions. Our results establish that combined MD simulation and experimental measurements offers a powerful strategy to define the structural elements underlying the PDZ-ligand interaction and advance the molecular-level understanding of PDZ domain specificity.

## Materials and Methods

### Model preparation and MD simulation

The 22-residue carboxy-terminal fragment of NPT2A (-ELPPATPSPRLALPAHHNATRL) was built using the Leap program (AMBER 9 [33]) (see Supporting Information for details). To model the pose of the NPT2A peptide in PDZ1, we used the PDZ1-NATRL complex from our prior MD simulation study [24] as a template. After superposing the carboxy-terminal motif of the–NATRL (22-residue peptide) over the carboxy-terminal motif of the–NATRL peptide (using backbone atoms), the short peptide was removed from the system. The final system includes PDZ1 and the 22-residue carboxy-terminal NPT2A motif (PDZ1-NPT2A complex). By convention, the carboxy-terminal residue is numbered starting at zero with upstream residues designated as -1, -2, -3, -4 etc. (Fig 2A). His residues in the PDZ1-NPT2A complex were treated as neutral by protonation at Nδ^1^.The PDZ1-NPT2A complex was solvated with TIP3P water molecules in a periodically replicated box, neutralized with a chloride ion and energy minimized over 500 steps including 100 steps of steepest descent minimization using the sander module of AMBER 9 [33]. Then equilibration and production simulations were run along 25 ns and 100 ns, respectively (see Supporting Information for details). As could be expected, N-terminal end (residues at position -7 to -21) of NPT2A does not reach a stable conformation at the end of the simulation at 125 ns and are not included for further analysis. MD trajectories obtained after equilibration were used for calculation non-covalent interactions (hydrogen bonds, salt bridges and hydrophobic contacts) [24,34] between PDZ1 and the bound NPT2A ligand as well as between PDZ2, a double PDZ2 mutant and NPT2A (see Supporting Information for details). We used the geometrical criteria (the donor-acceptor distance, donor-hydrogen-acceptor distance and the donor-hydrogen-acceptor angle) [10,24,34–37] to identify hydrogen bonds, salt bridges and hydrophobic contacts between PDZ1 and NPT2A.

The next series of MD simulations were performed for a mutant PDZ1-NPT2A complex. A double substitution of alanine residues for histidine residues at position -5 and -6 of the bound NPT2A peptide was computationally performed (His^-5^His^-6^/Ala^-5^Ala^-6^) using the Leap module AMBER 9 [33]. Energy minimization of the system performed by conjugate gradient method was followed by 20 ns and 50 ns equilibration and production MD simulations, respectively.

The initial PDZ2-NPT2A complex was generated using the PDZ1-NPT2A structure as a template. The initial coordinates for the PDZ2 domain were taken from our prior MD simulation study [24]. PDZ2 was overlaid with the PDZ1-NPT2A complex using the protein backbone atoms. After that the coordinates of PDZ1 were removed. The final complex includes the PDZ domain with the bound NPT2A peptide ligand. Based on this model, a double mutant PDZ2 with the bound NPT2A peptide was generated by a substitution of His for Asn167 (Asn167His) and Asp by Glu183 (Asp183Glu) using the Leap module of AMBER 9 [33]. Both systems were solvated with TIP3P water molecules in a periodically replicated box and neutralized with a chloride ion. The simulation set up for WT PDZ2 and the double PDZ2 mutant with the bound NPT2A peptide, as well as a protocol for energy minimization, equilibration and production simulations was similar as those for WT PDZ1-NPT2A (see Supporting Information for details). Equilibration and production simulations were run along 20 ns and 130 ns, respectively.

### Expression and purification of wild-type and mutant NHERF1

Plasmids for PDZ1 (11–120), His27Asn, and Glu43Asp were previously described [22]. The expression plasmids pET16-N1P1 encoding PDZ1 (1–140) and pET16-N1P2 encoding PDZ2 (133–300) of NHERF1 were kindly provided by Dr. Dale F. Mierke (Department of Chemistry, Dartmouth College, Hanover, NH, USA). The Asp183Glu and Asn167His/Asp183Glu mutations were introduced into pET16-N1P2 using the QuickChange mutagenesis kit (Stratagene) in order to generate the single and double mutant PDZ2. Plasmid fidelity was confirmed by DNA sequencing (ABI PRISM 377, Applied Biosystems, Foster City, CA) and subsequent sequence alignment (NCBI BLAST) with human NHERF1 (GenBank AF015926) to ensure the accuracy of the constructs. The recombinant proteins were expressed in *E*. *coli* BL21 (DE3) cells (Novagen) and purified using Ni-NTA-agarose (Qiagen) [28]. The resulting proteins were divided into aliquots and stored in phosphate buffer (25 mM NaH_2_PO_4_, 10 mM NaCl, pH 7.4) at -80°C until used for FP experiments.

### Peptide synthesis

The 22-residue NPT2A peptide was synthesized by solid phase methodology using standard Fmoc (N-(9-fluorenyl)methoxycarbonyl) chemistry (0.1 mmol scale) on an Applied Biosystems AB433 peptide synthesizer. After synthesis, the peptidyl resin was treated overnight with 4 eq of 5-(and 6)-carboxytetramethylrhodamine in the presence of HBTU/HOBt/DIEA. Following standard trifluoroacetic acid cleavage, the product was purified by HPLC on a Vydac C-18 reverse phase column and lyophilized. The final product was characterized by electron spray mass spectrometry. The rhodamine-labeled peptide was dissolved in acetic acid (0.1%). Peptide concentration was determined from the molar extinction coefficient for rhodamine. Then the rhodamine-labeled NPT2A peptide was serially diluted in storage buffer (25 mM NaH_2_PO_4_, 10 mM NaCl, pH 7.4).

### Fluorescence Polarization (FP) saturation binding assay

A solution phase direct binding assay was used to characterize the affinity of NHERF1 constructs to fluorescently labeled peptides [38]. FP measurements were performed following the protocol described by Madden and co-workers [10]. All measurements were performed in FP buffer (storage buffer, supplemented to a final concentration of 1 mM DTT, 0.1 mg/ml bovine IgG (Sigma) and 0.5 mM Thesit (Fluka) containing 0.5 μM or 1 μM fluorescent peptide for WT or mutant systems, respectively. Polarized fluorescence intensities were measured at 25°C with a Perkin Elmer Wallac Victor3 multilabel plate reader using excitation and emission wavelengths of 544 nm and 595 nm for the rhodamine-labeled peptide. FP assays were run in triplicate, with error bars representing the standard deviation. All measurements are reported as fluorescent anisotropy rather than polarization. Anisotropy was calculated using Eq 1 from the measured fluorescence emission intensities that are polarized parallel (I_∥_) and perpendicular (I) to the plane of the incident light [39]:

$$r=I||-\frac{I⊥}{I||}\;+2I⊥$$ (1)

The equilibrium dissociation constant (*K*_*D*_) for interaction between PDZ domain and peptide was determined by fitting the fluorescent anisotropy data to Eq 2 by non-linear regression analysis and assuming formation of a 1:1 complex [39].

$$A\;=A_{0}+\frac{K_{d}\;+\left[L\right]\;+\left[PDZ\right]-\;\sqrt{{\left(K_{d}\;+\left[L\right]\;+\left[PDZ\right]\right)}^{2}-\;4\left[L\right]\left[PDZ\right]}}{2\left[L\right]}\;\left(A_{m}\;-A_{0}\right)$$ (2)

where, *A* is the measured anisotropy, [*L*] and [*PDZ*] are the total concentration of peptide ligand and PDZ construct, *A*_0_ and *A*_*m*_ are low and upper anisotropy. All calculations were performed using Prism (GraphPad).

### Isothermal Titration Calorimetry (ITC)

ITC measurements were performed with a MicroCalTM Auto-iTC200 system (GE Healthcare) at 25°C. Before measurement, samples were dialyzed overnight at 4°C in a buffer containing 10 mM Hepes (pH 7.5), 10 mM NaCl, 0.5 mM EDTA, and 0.5 mM ββ-mercaptoethanol. For determining NPT2A binding to PDZ constructs, the reaction cells were filled with 460 μl of 25 μM of the indicated PDZ protein. The ligand NPT2A (200–250 μM), was titrated into the cell in 19 injections of 2 μl each, with 150 s intervals between each injection. To remove the contribution of NPT2A dilution heat, a control experiment has been performed by titrating NPT2A into the buffer, which was then subtracted from the actual experimental data.

The ITC data were analyzed using Microcal Origin 7.0 and the manufacturer's provided VPViewer module to yield the association constant (*Ka*), stoichiometry (n), and the observed enthalpy change (Δ*H*°) for the binding reactions. Analysis of ITC data directly yielded Δ*H*° and *Ka*. The Gibbs energy calculated using the equation:

ΔG°=−RTlnKa (3)

The entropy change was then obtained using the standard thermodynamic expression.

ΔG°=ΔH°−TΔS° (4)

## Supporting Information

S1 Fig The binding pocket of PDZ1. Overlay of the two PDZ1 structures, illustrating similar orientation of side chains involving in canonical interactions with target ligands. The light blue structure corresponds to the X-ray structure of PDZ1 (PDB code: 1GQ4, PDZ1-DSLL complex). The cyan structure corresponds to the average structure of PDZ1 from MD simulation (PDZ1-NPT2A complex). Overlay was performed using the Cα backbone atoms (residues 13–91). Peptide ligands are not shown in the PDZ1 binding site for simplicity.
(PDF)

S2 Fig Change in RMSFs of PDZ1 upon the NPT2A binding. The RMSF values of the Cα atoms of PDZ1 (black) and the PDZ1 bound to NPT2A (blue) with respect to the starting structure are presented.
(PDF)

S3 Fig Superimposing of structures of PDZ1 and PDZ2. Superimposing of the PDZ1 (cyan) and PDZ2 domains (pink) in complex with the NPT2A peptide (left). Key residues of PDZ1 involved in the interaction with NPT2A and corresponding residues in PDZ2 are shown in stick representation (right). Key differences are Glu43 and His27 in PDZ1 and Asp183 and Asn167 in PDZ2. The NPT2A peptide is not shown for simplicity. Hydrogen atoms are white, oxygens are red, and nitrogens are blue.
(PDF)

S4 Fig Superimposing of structures of PDZ1 and the double PDZ2 mutant. Superimposing of the PDZ1 domain (cyan) and the double PDZ2 mutant (Asn167His/Asp183Glu) (wheat) in complex with the NPT2A peptide (left). Stick representation of key residues of PDZ1 and the double PDZ2 mutant that rescues the interaction with the NPT2A peptide (right). The NPT2A peptide is not shown for simplicity. Atoms are colored as described in the legend to S3 Fig.
(PDF)

S5 Fig A representation of the structure of the double PDZ2 mutant-NPT2A complex. The NPT2A peptide is shown (wheat) within the canonical binding pocket between the α2-helix and β2-strand of the double PDZ2 mutant (Asn167His/Asp183Glu) (grey). The last five carboxy-terminal residues of the NPT2A peptide are shown in stick representation. Electrostatic interactions between the carboxylate group of Asp183Glu and Arg^-1^ of NPT2A as well as carbon-carbon interactions between Asn167His and Arg^-1^ are shown as black dotted lines. Atoms are colored as described in the legend to S3 Fig.
(PDF)

S6 Fig Interactions between the double PDZ2 mutant and NPT2A. **S6A Fig.** The evolution of an electrostatic interaction between Asp183Glu.Oε^1^ and Arg^-1^.NHη^21^ along the last 5 ns of MD simulation of the double PDZ2 mutant (Asn167His/Asp183Glu) in complex with the NPT2A peptide. **S6B Fig.** A salt bridge between the NHη^22^ group of Agr^-1^ and the carboxylate group of Asp183Glu is shown as a black dotted line (2.0Å). The 4.5Å carbon-carbon distance between the Cδ^2^ atom of Asn167His and the Cβ atom of Arg^-1^ and the Cε^1^ atom of Asn167His and the C atom of Arg^-1^ is shown as a black dotted line. Asp183 (green) and Asn167 (green) of WT PDZ2 do not form interactions with Arg^-1^ of NPT2A (green). Atoms are colored as described in the legend to S3 Fig.
(PDF)

S7 Fig His^-5^/Ala^-5^ and His^-6^/Ala^-6^ mutations in NPT2A destabilize the PDZ1-NPT2A complex. The RMSF values of the Cα atoms of PDZ1 (A) and the NPT2A peptide (B) with respect to the initial structure are presented.
(PDF)

S1 References Supporting references. (DOCX)

S1 Table Analysis of PDZ1-NPT2A Interactions. (DOCX)

S2 Table Empirical values of pK_a_ calculated by PROPKA3.1. (DOCX)

S1 Text NPT2A Peptide Model Preparation. (DOCX)

S2 Text MD Simulation. S2.1 The WT PDZ1-NPT2A complex. S2.2 The double PDZ2 mutant (Asp183Glu/Asn167His) with the limited-NATRL sequence of NPT2A.
(DOCX)

S3 Text Analysis of MD Trajectories. (DOCX)
