# Supplementary material for: Canonical and Noncanonical Sites Determine NPT2A Binding Selectivity to NHERF1 PDZ1
Source: PLoS One. 2015 Jun 12;10(6):e0129554. doi: 10.1371/journal.pone.0129554 (PMC4466390; doi:10.1371/journal.pone.0129554)

Supporting Information Figure S1

The binding pocket of PDZ1.

Overlay of the two PDZ1 structures, illustrating similar orientation of side chains involving in canonical interactions with target ligands. The light blue structure corresponds to the X-ray structure of PDZ1 (PDB code: 1GQ4, PDZ1-DSLL complex). The cyan structure corresponds to the average structure of PDZ1 from MD simulation (PDZ1-NPT2A complex). Overlay was performed using the Cα backbone atoms (residues 13-91). Peptide ligands are not shown in the PDZ1 binding site for simplicity.

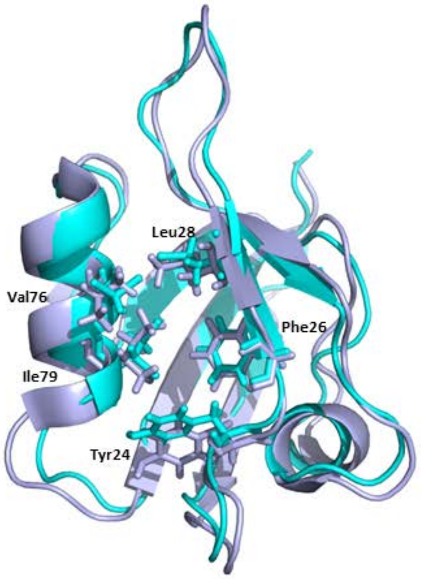

Supplement: S1 Fig — Overlay of the two PDZ1 structures, illustrating similar orientation of side chains involving in canonical interactions with target ligands. The light blue structure corresponds to the X-ray structure of PDZ1 (PDB code: 1GQ4, PDZ1-DSLL complex). The cyan structure corresponds to the average structure of PDZ1 from MD simulation (PDZ1-NPT2A complex). Overlay was performed using the Cα backbone atoms (residues 13–91). Peptide ligands are not shown in the PDZ1 binding site for simplicity. (PDF) [file pone.0129554.s001.pdf]
