# Supplementary material for: Canonical and Noncanonical Sites Determine NPT2A Binding Selectivity to NHERF1 PDZ1
Source: PLoS One. 2015 Jun 12;10(6):e0129554. doi: 10.1371/journal.pone.0129554 (PMC4466390; doi:10.1371/journal.pone.0129554)

Supporting Information Figure S2

Change in RMSFs of PDZ1 upon the NPT2A binding.

The RMSF values of the Cα atoms of PDZ1 (black) and the PDZ1 bound to NPT2A (blue) with respect to the starting structure are presented.

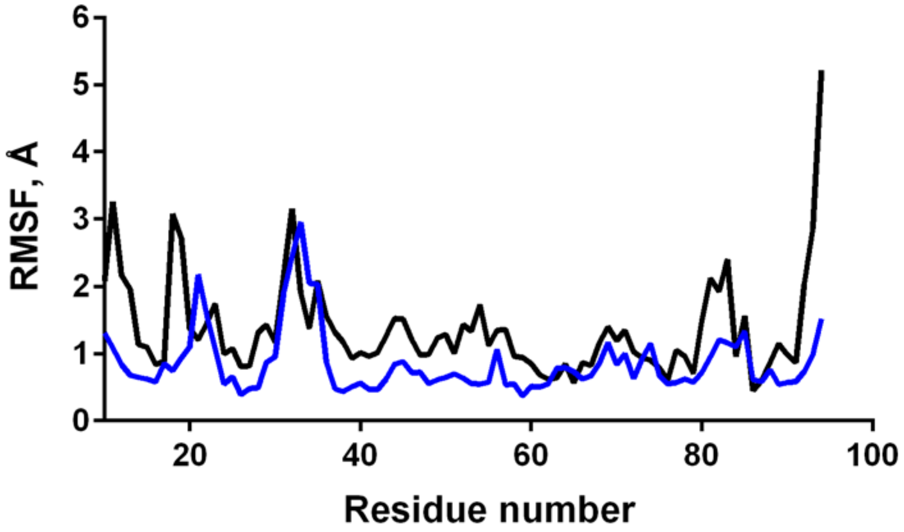

Supplement: S2 Fig — The RMSF values of the Cα atoms of PDZ1 (black) and the PDZ1 bound to NPT2A (blue) with respect to the starting structure are presented. (PDF) [file pone.0129554.s002.pdf]
