# Supplementary material for: Canonical and Noncanonical Sites Determine NPT2A Binding Selectivity to NHERF1 PDZ1
Source: PLoS One. 2015 Jun 12;10(6):e0129554. doi: 10.1371/journal.pone.0129554 (PMC4466390; doi:10.1371/journal.pone.0129554)

Supporting Information Figure S3

Superimposing of structures of PDZ1 and PDZ2.

Superimposing of the PDZ1 (cyan) and PDZ2 domains (pink) in complex with the NPT2A peptide (left). Key residues of PDZ1 involved in the interaction with NPT2A and corresponding residues in PDZ2 are shown in stick representation (right). Key differences are Glu43 and His27 in PDZ1 and Asp183 and Asn167 in PDZ2. The NPT2A peptide is not shown for simplicity. Hydrogen atoms are white, oxygens are red, and nitrogens are blue.

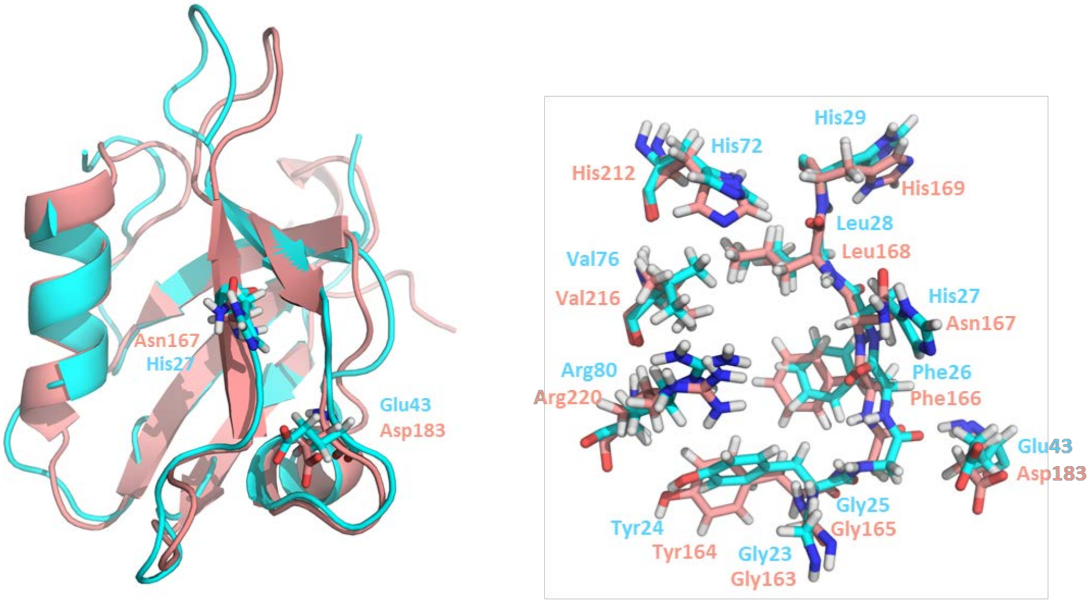

Supplement: S3 Fig — Superimposing of the PDZ1 (cyan) and PDZ2 domains (pink) in complex with the NPT2A peptide (left). Key residues of PDZ1 involved in the interaction with NPT2A and corresponding residues in PDZ2 are shown in stick representation (right). Key differences are Glu43 and His27 in PDZ1 and Asp183 and Asn167 in PDZ2. The NPT2A peptide is not shown for simplicity. Hydrogen atoms are white, oxygens are red, and nitrogens are blue. (PDF) [file pone.0129554.s003.pdf]
