# Supplementary material for: Canonical and Noncanonical Sites Determine NPT2A Binding Selectivity to NHERF1 PDZ1
Source: PLoS One. 2015 Jun 12;10(6):e0129554. doi: 10.1371/journal.pone.0129554 (PMC4466390; doi:10.1371/journal.pone.0129554)

Supporting Information Figure S4

Superimposing of structures of PDZ1 and the double PDZ2 mutant.

Superimposing of the PDZ1 domain (cyan) and the double PDZ2 mutant (Asn167His/Asp183Glu) (wheat) in complex with the NPT2A peptide (left). Stick representation of key residues of PDZ1 and the double PDZ2 mutant that rescues the interaction with the NPT2A peptide (right). The NPT2A peptide is not shown for simplicity. Atoms are colored as described in the legend to S3A Fig.

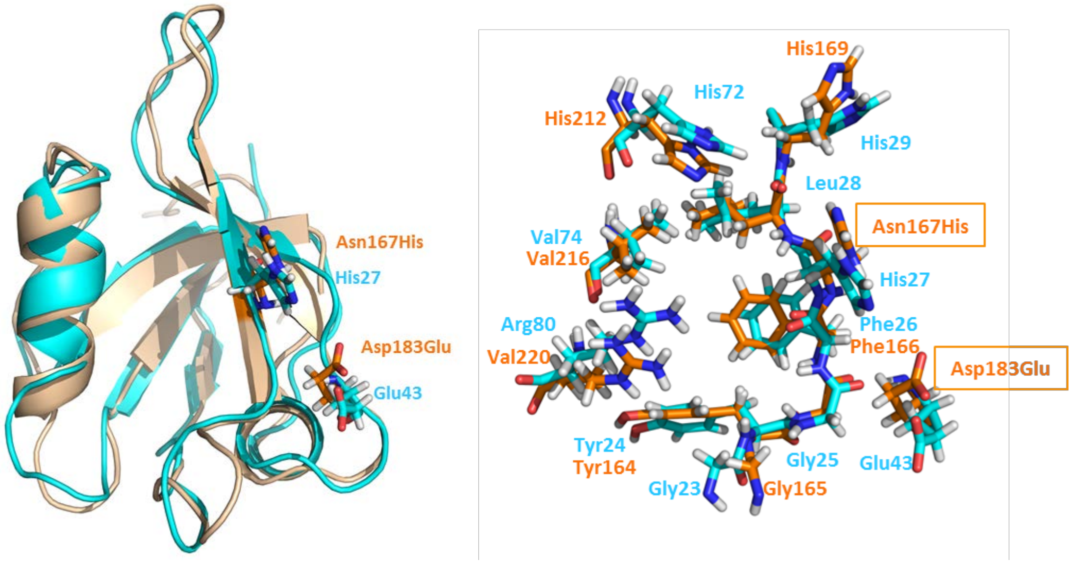

Supplement: S4 Fig — Superimposing of the PDZ1 domain (cyan) and the double PDZ2 mutant (Asn167His/Asp183Glu) (wheat) in complex with the NPT2A peptide (left). Stick representation of key residues of PDZ1 and the double PDZ2 mutant that rescues the interaction with the NPT2A peptide (right). The NPT2A peptide is not shown for simplicity. Atoms are colored as described in the legend to S3 Fig. (PDF) [file pone.0129554.s004.pdf]
