# Supplementary material for: Canonical and Noncanonical Sites Determine NPT2A Binding Selectivity to NHERF1 PDZ1
Source: PLoS One. 2015 Jun 12;10(6):e0129554. doi: 10.1371/journal.pone.0129554 (PMC4466390; doi:10.1371/journal.pone.0129554)

Supporting Information Figure S5

A representation of the structure of double PDZ2 mutant-NPT2A complex.

The NPT2A peptide is shown (wheat) within the canonical binding pocket between the α2-helix and β2–strand of the double PDZ2 mutant (Asn167His/Asp183Glu) (grey). The last five carboxy-terminal residues of the NPT2A peptide are shown in stick representation. Electrostatic interactions between the carboxylate group of Asp183Glu and Arg^{-1} of NPT2A as well as carbon-carbon interactions between Asn167His and Arg^{-1} are shown as black dotted lines. Atoms are colored as described in the legend to S3A Fig.

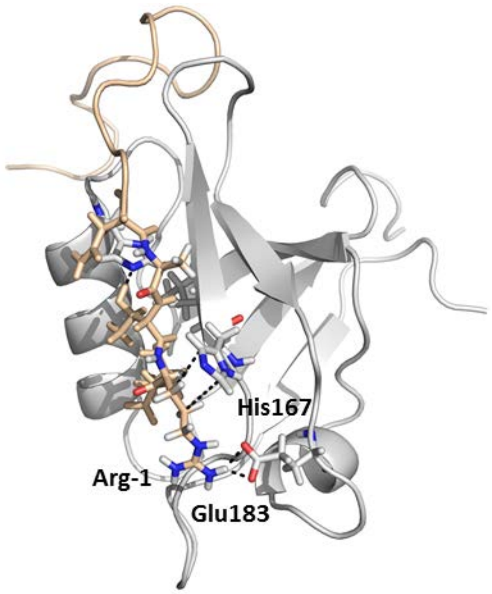

Supplement: S5 Fig — The NPT2A peptide is shown (wheat) within the canonical binding pocket between the α2-helix and β2-strand of the double PDZ2 mutant (Asn167His/Asp183Glu) (grey). The last five carboxy-terminal residues of the NPT2A peptide are shown in stick representation. Electrostatic interactions between the carboxylate group of Asp183Glu and Arg-1 of NPT2A as well as carbon-carbon interactions between Asn167His and Arg-1 are shown as black dotted lines. Atoms are colored as described in the legend to S3 Fig. (PDF) [file pone.0129554.s005.pdf]
