# Supplementary material for: Canonical and Noncanonical Sites Determine NPT2A Binding Selectivity to NHERF1 PDZ1
Source: PLoS One. 2015 Jun 12;10(6):e0129554. doi: 10.1371/journal.pone.0129554 (PMC4466390; doi:10.1371/journal.pone.0129554)
Supplement: S6 Fig — S6A Fig. The evolution of an electrostatic interaction between Asp183Glu.Oε1 and Arg-1.NHη21 along the last 5 ns of MD simulation of the double PDZ2 mutant (Asn167His/Asp183Glu) in complex with the NPT2A peptide. S6B Fig. A salt bridge between the NHη22 group of Agr-1 and the carboxylate group of Asp183Glu is shown as a black dotted line (2.0Å). The 4.5Å carbon-carbon distance between the Cδ2 atom of Asn167His and the Cβ atom of Arg-1 and the Cε1 atom of Asn167His and the C atom of Arg-1 is shown as a black dotted line. Asp183 (green) and Asn167 (green) of WT PDZ2 do not form interactions with Arg-1 of NPT2A (green). Atoms are colored as described in the legend to S3 Fig. (PDF) [file pone.0129554.s006.pdf]

Supporting Information Figure S6

Interactions between the double PDZ2 mutant and NPT2A.

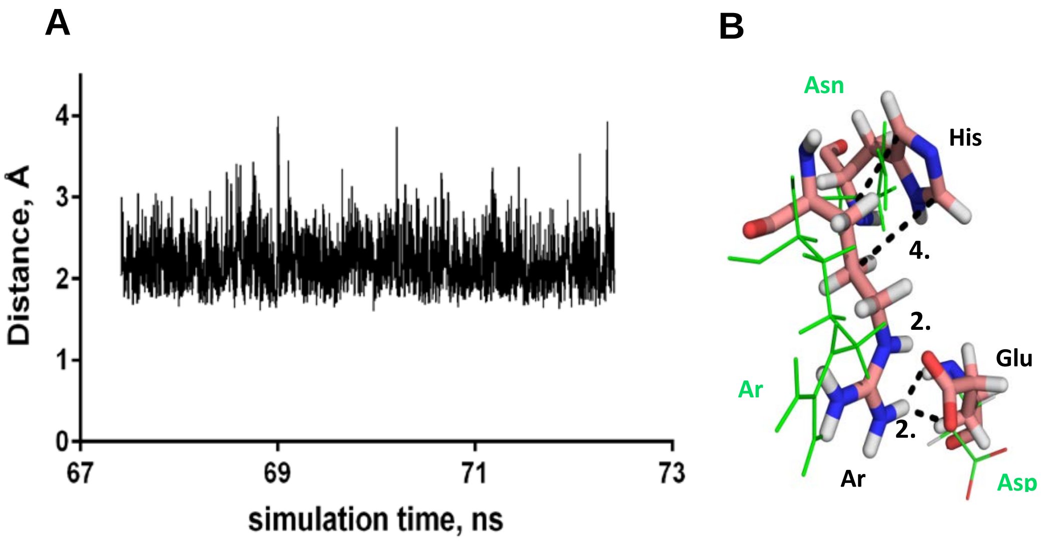

(A) The evolution of an electrostatic interaction between Asp183Glu.Oε^1 and Arg^{-1}.NHη^{21} along the last 5 ns of MD simulation of the double PDZ2 mutant (Asn167His/Asp183Glu) in complex with the NPT2A peptide. **(B)** A salt bridge between the NHη^{22} group of Agr^{-1} and the carboxylate group of Asp183Glu is shown as a black dotted line (2.0Å). The 4.5 Å carbon-carbon distance between the Cδ^2 atom of Asn167His and the Cβ atom of Arg^{-1} and the Cε^1 atom of Asn167His and the Cγ atom of Arg^{-1} is shown as a black dotted line. Asp183 (green) and Asn167 (green) of WT PDZ2 do not form interactions with Arg^{-1} of NPT2A (green). Atoms are colored as described in the legend to S3A Figure.
