# Supplementary material for: Canonical and Noncanonical Sites Determine NPT2A Binding Selectivity to NHERF1 PDZ1
Source: PLoS One. 2015 Jun 12;10(6):e0129554. doi: 10.1371/journal.pone.0129554 (PMC4466390; doi:10.1371/journal.pone.0129554)

Supporting Information Figure S7

His^{-5}/Ala^{-5} and His^{-6}/Ala^{-6} mutations in NPT2A destabilize the PDZ1-NPT2A complex.

The RMSF values of the Cα atoms of PDZ1 **(A)** and the NPT2A peptide **(B)** with respect to the initial structure are presented.

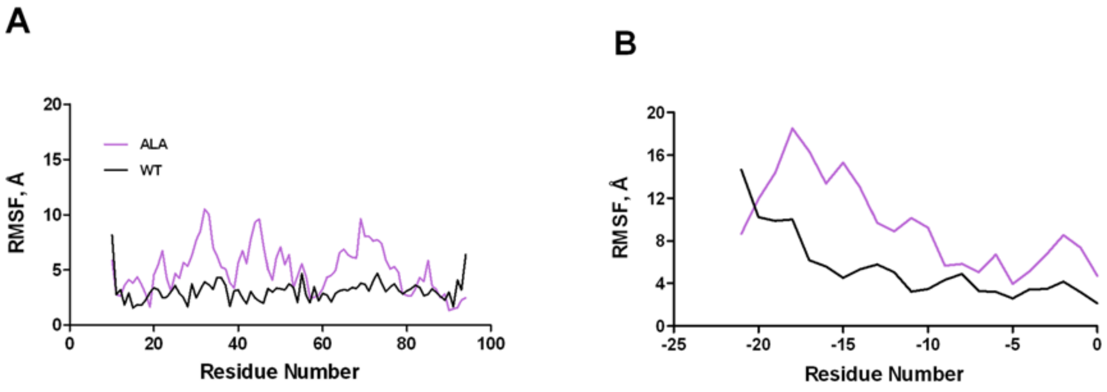

Supplement: S7 Fig — The RMSF values of the Cα atoms of PDZ1 (A) and the NPT2A peptide (B) with respect to the initial structure are presented. (PDF) [file pone.0129554.s007.pdf]
