# Supplementary material for: Canonical and Noncanonical Sites Determine NPT2A Binding Selectivity to NHERF1 PDZ1
Source: PLoS One. 2015 Jun 12;10(6):e0129554. doi: 10.1371/journal.pone.0129554 (PMC4466390; doi:10.1371/journal.pone.0129554)
Supplement: S1 References — (DOCX) [file pone.0129554.s008.docx]

### Supporting References

Case, D.A., Darden T.A,Cheatham, III, C.L. Simmerling, J. Wang, R.E. Duke, R., Luo, K.M.M., D.A. Pearlman, M. Crowley, R.C. Walker, W. Zhang, B. Wang, S., Hayik, A.R., G. Seabra, K.F. Wong, F. Paesani, X. Wu, S. Brozell, V. Tsui, H., Gohlke, L.Y., C. Tan, J. Mongan, V. Hornak, G. Cui, P. Beroza, D.H. Mathews, C., and Schafmeister, W.S.R., and P.A. Kollman (2006). AMBER 9.

Darden, T., York, D., and Pedersen, L. (1993). Particle mesh Ewald - an N•log(N) method for Ewald sums in large systems. J. Chem. Phys. *98*, 10089-10092.

Berendsen, H.J.C., Postma, J.P.M., Vangunsteren, W.F., Dinola, A., and Haak, J.R. (1984). Molecular dynamics with coupling to an external bath. J. Chem. Phys. *81*, 3684-3690.

Mamonova, T., Kurnikova, M., and Friedman, P.A. (2012). Structural Basis for NHERF1 PDZ Domain Binding. Biochemistry *51*, 3110-3120.

Hornak, V., Abel, R., Okur, A., Strockbine, B., Roitberg, A., and Simmerling, C. (2006). Comparison of multiple amber force fields and development of improved protein backbone parameters. Proteins:Struct. Funct. Bioinform. *65*, 712-725.

Kurnikov, I. V. HAmiltonian to Research LargE Molecules (HARLEM). Available:

http://harlem.chem.cmu.edu/.

Mamonova, T., Glyakina A.V., Galzitskaya O.V., amd Kurnikova M.G. (2013) Stability and rigidity/flexibility-Two sides of the same coin? Biochem. Biopys. Acta. ProteinsProteomics.1834, 854-866.
