# Supplementary material for: Canonical and Noncanonical Sites Determine NPT2A Binding Selectivity to NHERF1 PDZ1
Source: PLoS One. 2015 Jun 12;10(6):e0129554. doi: 10.1371/journal.pone.0129554 (PMC4466390; doi:10.1371/journal.pone.0129554)
Supplement: S1 Table — (DOCX) [file pone.0129554.s009.docx]

**Supporting Information Table S1**

**Table S1. Analysis of PDZ1-NPT2A Interactions**^a^

| \| Donor-Hydrogen pair \| Acceptor \| \| --- \| --- \| \| Gly30.N-H  Leu^0^.N-H  Leu^0^.O  Arg80.Nη2-Hη21  Thr^-2^.Oγ1-Hγ1  Arg80.Nε-Hε  His27.N-H  His27.Nδ1-Hδ1  Arg40.N-H  Ser46.Oγ-Hγ  His29.N-H  Tyr38.N-H  Leu28.N-H  Thr^-2^.N-H  Val76.N-H  His.72Nδ1-Hδ1  Arg^-1^.Nε-Hε  Arg^-1^.Nε-Hε  Arg^-1^.Nη2-Hη21  Arg^-1^.Nη2-Hη21 \| His^-6^.O  Phe26.O  Tyr24.N-H  Arg^-1^.O  His72.Nε2  Leu^0^.O  Arg40.O  Ala^-3^.O  His27.O  Glu43.O  Tyr38.O  His29.O  Thr^-2^.O  Leu28.O  His72.O  Gln37.Oε1  Glu43.Oε2  Glu43.Oε1  Glu43.Oε2  Glu43.Oε1 \| | \| Hydrophobic \| Interactions \| \| --- \| --- \| \| His^-6^.Cε1  His^-6^.Cγ  Leu^0^.Cδ2  Arg^-1^.Cγ  Arg^-1^.Cβ  Thr^-2^.Cβ  Leu^0^.Cδ1  Leu^0^.Cδ2  Leu^0^.Cβ  Leu^0^.Cβ  His^-6^.Cβ  Leu17.Cδ2  Leu^0^.Cδ1  His^-6^.Cδ2  His^-6^.Cγ  Leu^0^.Cδ2  His^-6^.Cβ  His27.Cδ2  Arg^-1^.Cβ  His29.Cβ  His^-5^. Cγ  His^-5^.Cδ2 \| His72.Cγ  His72.Cδ2  Phe26.Cδ2  His27.Cε1^b^  His27. Cδ2^b^  His72.Cε1  Ile79.Cγ2  Leu28.Cβ  Tyr24.Cδ1  Tyr24.Cδ1  His72.Cε1  Phe26.Cε1  Val76.Cγ1  His72.Cδ2  His72.Cγ  Phe26.Cε2  His72.Cβ  Leu41.Cβ  His27.Cε1  Arg40.Cγ  His29. Cγ^b^  His29.Cε1^b^ \| |
| --- | --- | --- | --- | --- | --- | --- | --- | --- | --- |

^a^ Interactions persisting more than 50% along a 20 ns MD trajectory are included.

^b^The C-C distance varies between 4-5Å
