# Supplementary material for: Canonical and Noncanonical Sites Determine NPT2A Binding Selectivity to NHERF1 PDZ1
Source: PLoS One. 2015 Jun 12;10(6):e0129554. doi: 10.1371/journal.pone.0129554 (PMC4466390; doi:10.1371/journal.pone.0129554)
Supplement: S2 Table — (DOCX) [file pone.0129554.s010.docx]

**Supporting Information Table S2**

**Table S2. Empirical values of pK_a_ calculated by PROPKA3.1**

| Residue | pK_a_ |
| --- | --- |
| His^-5^  His^-6^  His27  His29  His72 | 6.11  5.83  6.49  5.98  4.90 |
