# Supplementary material for: Canonical and Noncanonical Sites Determine NPT2A Binding Selectivity to NHERF1 PDZ1
Source: PLoS One. 2015 Jun 12;10(6):e0129554. doi: 10.1371/journal.pone.0129554 (PMC4466390; doi:10.1371/journal.pone.0129554)
Supplement: S1 Text — (DOCX) [file pone.0129554.s011.docx]

### S1 Text. NPT2A Peptide Model Preparation.

The 22-residue carboxy-terminal fragment of NPT2A (‑ELPPATPSPRLALPAHHNATRL) was built using the Leap program (AMBER 9 ([Case et al., 2006](#_ENREF_2)). N- and C-terminal ends of the peptide ligands were modeled as NH_3_^+^ and COO^-^, respectively. The peptide was solvated with TIP3P water (AMBER 9, ([Case et al., 2006](#_ENREF_2))) in a rectangular box. A 200-step energy minimization was performed using the SANDER module of AMBER 9 ([Case et al., 2006](#_ENREF_2)). The peptide was equilibrated for 200 ps (NVT ensemble). Then the initial conformation of the peptide was chosen randomly from a 200 ps MD trajectory.
