# Supplementary material for: Canonical and Noncanonical Sites Determine NPT2A Binding Selectivity to NHERF1 PDZ1
Source: PLoS One. 2015 Jun 12;10(6):e0129554. doi: 10.1371/journal.pone.0129554 (PMC4466390; doi:10.1371/journal.pone.0129554)
Supplement: S2 Text — S2.1 The WT PDZ1-NPT2A complex. S2.2 The double PDZ2 mutant (Asp183Glu/Asn167His) with the limited-NATRL sequence of NPT2A. (DOCX) [file pone.0129554.s012.docx]

**S2 Text. MD Simulation.**

**S2.1 The WT PDZ1-NPT2A complex.**

MD simulations were performed using the pmemd module of AMBER 11 with the ff94 force fields. Periodic boundary conditions were applied, and the Particle Mesh Ewald method ([Darden et al., 1993](#_ENREF_3)) was used use to compute long-range electrostatic and Lennard-Jones interactions with 12Å. Pressure was kept at 1 atm using a Beredsen barostat ([Berendsen et al., 1984](#_ENREF_1)), and temperature was held at 300 K using the Berendsen thermostat. Weak harmonic restraints (*k_s_* = 0.1 kcal/(mol**^.^**Å^2^)) were applied to the peptide N-terminal and C-terminal backbone atoms to prevent diffusion. Dimensions of the simulation box before equilibration was 58x67x60Å. Equilibration and production runs of 25 ns and 100 ns, respectively, were carried out in the canonical NVT ensemble with configurations saved every 2 fs for analysis. The last 20 ns of the MD trajectory were considered for analysis.

**S2.2 The double PDZ2 mutant (Asp183Glu/Asn167His) with the limited -NATRL sequence of NPT2A**.

The coordinates of the PDZ2 domain were taken from our prior simulation study ([Mamonova et al., 2012](#_ENREF_6)). Using the Leap module AMBER 9 ([Case et al., 2006](#_ENREF_2)) computational modification was performed in PDZ2. Histidine was substituted for Asn167, and glutamic acid was substituted for aspartic acid 183. PDZ2 was overlaid with the PDZ1-NATRL complex ([Mamonova et al., 2012](#_ENREF_6)) using backbone atoms, and then PDZ1 was removed. The double PDZ2 mutant with the bound –NATRL peptide was solvated using the TIP3P water model in a rectangular box. A chloride ion was added to neutralize the system. The solvated system was minimized during 200 steps using the steepest descent and conjugate gradient methods (SANDER module of AMBER 9 ([Case et al., 2006](#_ENREF_2))). Equilibration and production runs of 20 ns and 50 ns simulations, respectively, were carried out in the NVT ensemble with weak harmonic restrains (*k_s_* = 0.1 kcal/(mol**^.^**Å^2^)) applied to the N-terminal backbone atoms of the peptide as well as to the protein C-terminal backbone atoms to prevent diffusion. Explicit solvent MD simulation carried out using the AMBER 11 PMEMD program and an ff99SB force field ([Hornak et al., 2006](#_ENREF_4)).
