# Supplementary material for: Canonical and Noncanonical Sites Determine NPT2A Binding Selectivity to NHERF1 PDZ1
Source: PLoS One. 2015 Jun 12;10(6):e0129554. doi: 10.1371/journal.pone.0129554 (PMC4466390; doi:10.1371/journal.pone.0129554)
Supplement: S3 Text — (DOCX) [file pone.0129554.s013.docx]

**S3 Text. Analysis of MD Trajectories.**

For each system the root-mean-square deviation (RMSD) of the Cα atoms of PDZ1 (residues 13-91, excluding three N- and C-terminal residues), PDZ2, or double PDZ2 mutant (residues 151-236, excluding three N- and C-terminal residues) relative to the starting structure was calculated and used to assess the system equilibration. The RMSD values of the backbone atoms for the five carboxy-terminal residues of the NPT2A peptide (position 0 to -4) were used to characterize the stability of the bound peptide in the binding site. To compute relative mobility of the protein as well as ligand residues, the root mean square fluctuation (RMSF) of each Cα as a function of residue number was calculated with respect to the starting structure. Because subtle conformational changes were observed in the side chains of the peptide, as well as the residues lining the PDZ binding pocket, during the first approximately 40 ns of MD simulation, analysis of hydrogen bonds (HB), salt bridges (SB) and hydrophobic interactions (HP) was performed on 5 ns trajectories selected from the last 20 ns of each MD simulation to identify PDZ-ligand interactions. The MD trajectories were analyzed using PTRAJ module of AMBER package, HARLEM (HAmiltonian for Response properties of LargE Molecules) and Python scripts developed in house ([Kurnikov](#_ENREF_5)). The criteria used to identify HB, SB, or HP were taken from our earlier work ([Mamonova et al., 2012](#_ENREF_6), Mamonova et al., 2013).
